# 68. Impact of *Streptococcus pneumoniae* Urinary Antigen Testing in Patients with Community-Acquired Pneumonia Admitted within a Large Academic Medical System

**DOI:** 10.1093/ofid/ofab466.270

**Published:** 2021-12-04

**Authors:** Adam Greenfield, Kassandra L Marsh, Justin Siegfried, Ioannis Zacharioudakis, Nabeela Ahmed, Arnold Decano, Maria E Aguero-Rosenfeld, Kenneth Inglima, John Papadopoulos, Yanina Dubrovskaya

**Affiliations:** 1 NYU Langone Health, Yardley, Pennsylvania; 2 New York University Grossman School of Medicine, New York, New York; 3 NYU Langone - Brooklyn Hospital, Brooklyn, NY; 4 New York University, New York, NY

## Abstract

**Background:**

Limited data support the use of pneumococcal urinary antigen testing (PUAT) for patients admitted with community-acquired pneumonia (CAP) as a stewardship tool to curtail the use of broad-spectrum antimicrobials. At NYULH, CAP guidelines and admission order set were developed to standardize diagnostic testing, including PUAT. In this study we describe patients with positive versus negative PUAT and evaluate de-escalation and patients’ outcomes.

**Methods:**

This was a retrospective study of adults admitted with diagnosis of CAP between January-December 2019 who had a PUAT performed. The primary outcome was incidence and timing of de-escalation of antimicrobials following PUAT result. Among patients with a positive PUAT we compared hospital length of stay (LOS), incidence of Clostridioides difficile infection (CDI), infection-related readmission within 30 days, and in-hospital mortality among those who were de-escalated versus those who were not de-escalated/required escalation.

**Results:**

We evaluated 910 patients, of which 121 (13.3%) were PUAT positive. No difference in baseline characteristics, including severity of illness as represented by the Pneumonia Severity Index (97 [IQR 76-117] vs 89 [IQR 67-115], p=0.083) and Charlson Comorbidity Index, were observed between PUAT positive and negative groups. Time to PUAT testing occurred shortly after presentation to the hospital in both cohorts (16h [IQR 16-27] vs 13h [IQR 8-22], p=0.140). Initial de-escalation occurred in 97/117 (82.9%) and 629/775 (81.2%) of PUAT positive and negative patients, respectively (p = 0.749). Median time to de-escalation was shorter in the PUAT positive cohort (1 [IQR 0-2] vs 1 [IQR 1-2] day, p = 0.01). Among the PUAT positive group, hospital LOS stay was shorter in patients who were de-escalated compared to those who were not de-escalated/required escalation (6 days [IQR 4-10] vs 8 days [IQR 7-12], p=0.0005) with no difference in the incidence of CDI (2 [2.1%] vs 1 [3.7%], p=0.535), in-hospital mortality (4 [4.3%] vs 3 [11.1%], p=0.185), or 30-day infection-related readmission (2 [2.1%] vs 1 [3.7%], p=0.535).

**Conclusion:**

PUAT positivity resulted in quicker time to targeted therapy for CAP. Among patients with a positive PUAT, initial de-escalation of antimicrobials did not lead to worse patient outcomes.

**Disclosures:**

**All Authors**: No reported disclosures

